# Suramin: A Potential Therapy for Diabetic Nephropathy

**DOI:** 10.1371/journal.pone.0073655

**Published:** 2013-09-09

**Authors:** Midhun C. Korrapati, Lauren H. Howell, Brooke E. Shaner, Judit K. Megyesi, Leah J. Siskind, Rick G. Schnellmann

**Affiliations:** 1 Department of Drug Discovery and Biomedical Sciences, Medical University of South Carolina, Charleston, South Carolina, United States of America; 2 Department of Internal Medicine, College of Medicine, Division of Nephrology, University of Arkansas for Medical Sciences, Little Rock, Arkansas, United States of America; 3 Ralph H. Johnson Veterans Administration Medical Center, Charleston, South Carolina, United States of America; UAE University, Faculty of Medicine & Health Sciences, United Arab Emirates

## Abstract

**Objective:**

To determine whether delayed administration of a single dose of suramin, a drug that has been used extensively in humans to treat trypanosomiasis, attenuates renal injury in a leptin receptor deficient C57BLKS/J *db/db* type 2 diabetic nephropathy (T2DN) mouse model.

**Research Design and Methods:**

Groups of female non-diabetic (control) *db/m* and diabetic *db/db* mice of 8 and 16 weeks of age, respectively, were treated with suramin (10 mg/kg) or saline i.v. All animals were euthanized one week later. Measurements in mice 1 week following treatment included the following: body weight; blood glucose; urinary protein excretion; pathological lesions in glomeruli and proximal tubules; changes in protein expression of pro-inflammatory transcription factor nuclear factor κB (NF-κB) and intracellular adhesion molecule-1 (ICAM-1), profibrotic transforming growth factor-β1 (TGF-β1), phospho-SMAD-3 and alpha-smooth muscle actin (α-SMA); and immunohistochemical analysis of leukocyte infiltration and collagen 1A2 (COL1A2) deposition.

**Results:**

Immunoblot analysis revealed increased NF-κB, ICAM-1, TGF-β1, phospho-SMAD-3, and α-SMA proteins in both 9 and 17 week *db/db* mice as compared to *db/m* control mice. Immunohistochemical analysis revealed moderate leukocyte infiltration and collagen 1A2 (COL1A2) deposition in 9 week *db/db* mice that was increased in the 17 week *db/db* mice. Importantly, suramin significantly decreased expression of all these markers in 9 week *db/db* mice and partially decreased in 17 week *db/db* mice without altering body weight, blood glucose or urinary protein excretion. There was no difference in creatinine clearance between 9 week *db/m* and *db/db* mice ± suramin. Importantly, in the 17 week *db/db* mice suramin intervention reversed the impaired creatinine clearance and overt histological damage.

**Conclusions:**

Delayed administration of a single dose of suramin in a model of T2DN attenuated inflammation and fibrosis as well as improved renal function, supporting the use of suramin in T2DN.

## Introduction

Diabetic nephropathy (DN) is a clinically significant complication of diabetes and accounts for approximately 50% of all end-stage renal diseases (ESRD). This results in increasing renal replacement therapy and healthcare costs [1]. In addition, DN is a risk factor for cardiovascular disease [2], [3]. Although many pathophysiologic processes are involved in the pathogenesis of DN, the underlying mechanisms of DN are not fully established [4].

The three hallmark pathological features of DN are oxidative stress, inflammation and fibrosis, which mediate glomerular, interstitial and tubular damage [5], [6], [7]. Indeed, renal oxidative stress, inflammation and fibrosis are detected in murine models of DN and in kidneys of diabetic patients [8], [9], [10], suggesting that targeting these factors might be beneficial to therapeutic development. However, the renal protection provided by existing therapeutic modalities is insufficient to control the progression of DN, at least in part due to the late stage intervention that typically occurs with type 2 DN (T2DN) [11], [12]. Therefore, development of novel interventional strategies to blunt the progression of early as well as established DN to ESRD is essential.

Suramin, a drug that has been used extensively in humans to treat trypanosomiasis, was recently shown to accelerate recovery from acute kidney injury (AKI) by blunting the activation of pro-inflammatory mediators and expression of profibrotic factors following injury in mice and rat models [13], [14], [15]. It is important to note that suramin was administered after the kidney dysfunction and damage was established. Therefore, we sought to determine whether delayed administration of suramin suppresses inflammation and fibrosis and restores renal function in a T2DN model. To do this, we utilized *db/db* mice, a mouse T2DN model that appears to more closely mimic the progression of human DN [8]. A single dose of suramin was administered to 8 and 16 week old *db/db* mice, which represents early and late stage DN, respectively. The data revealed that delayed administration of suramin ameliorated inflammation and fibrosis in DN and improved renal function.

## Materials and Methods

### Animals and Treatment

Female diabetic *db/db* mice (BKS.Cg-m +/+ Lepr^db^/J; related genotype: a/a+Lepr^db^/+ Lepr^db^) and female non-diabetic *db/m* mice (BKS.Cg-m +/+ Lepr^db^/J; related genotype: a/a+Dock7^m^ +/+ Lepr^db^) were purchased from The Jackson Laboratory (Bar Harbor, ME; Stock Number: 000642) and were housed in temperature-controlled conditions under a light/dark photocycle with food and water supplied *ad libitum* at all times. At 8 (N = 11/each group) and 16 weeks (N = 6) of age *db/m* or *db/db* mice were injected with suramin (10 mg/kg), intravenously (i.v.) through the tail vein or saline (volume of injection ranged from 0.2–0.5 ml/mouse based on the body weights). One week later mice were placed in metabolism cages for 24 h and then euthanized.

Serum and urine were collected from all mice and kidneys harvested. One kidney was fixed in 4.5% buffered formalin, dehydrated, and embedded in paraffin. For general histology, sections were stained with PAS and scored in a blinded manner by Dr. Judit K. Megyesi. Unstained slides were used for immunohistochemical analysis. For immunohistochemistry of COL1A2, the manufacturer’s protocol was followed (Santa Cruz Biotechnology, Santa Cruz, CA). The other kidney was frozen at −80°C until further analysis. All animal and treatment protocols were in compliance with the Guide for Care and Use of Laboratory Animals as adopted and promulgated by the US National Institutes of Health and were approved by the Institutional Animal Care and Use Committee (IACUC) at the Medical University of South Carolina (MUSC). Body weights and serum glucose levels (BioAssay Systems, Hayward, CA) were determined. Urinary protein levels and volumes (24 hour urine collections were made on ice) were recorded and renal function was monitored by measuring serum and urine creatinine using a creatinine assay kit (BioAssay Systems, Hayward, CA) as per manufacturer’s instructions and creatinine clearance (CrCl) was calculated [15].

### Chemicals

Unless stated otherwise, all chemicals and reagents were purchased from Sigma Chemical Co. (St. Louis, MO). Sources of antibodies are listed below: 1) mouse monoclonal anti-transforming growth factor-β_1_ (TGF-β_1_) and rabbit polyclonal anti-intracellular adhesion molecule-1 (ICAM-1) - Abcam Inc. (Cambridge, MA), 2) goat polyclonal anti-collagen 1A2 - Santa Cruz Biotechnology (Santa Cruz, CA), 3) mouse monoclonal anti-alpha-smooth muscle actin - Sigma Chemical Co. (St. Louis, MO), 4) rabbit monoclonal anti-phospho-SMAD-3 and anti-phospho-p65 - Cell Signaling Technology (Beverly, MA), and 5) loading control glyceraldehyde 3-phosphate dehydrogenase (GAPDH) - Fitzgerald International Inc. (Acton, MA). Anti-goat secondary antibody conjugated with horseradish peroxidase was purchased from Millipore (Billerica, MA), and anti-rabbit and anti-mouse secondary antibodies conjugated with horseradish peroxidase were obtained from Pierce (Rockford, IL).

### Assessment of Renal Inflammation

Renal inflammation was assessed by measuring leukocyte (neutrophils, monocytes) infiltration using the naphthol AS-D chloroacetate esterase kit (Sigma Chemical Co., St. Louis, MO) and immunohistochemistry carried out as per manufacturer’s protocol. To quantify leukocyte infiltration, a total of 25 fields (original magnification x20) in the cortical region of the kidney sections were examined and expressed as the total number of leukocytes in all the fields.

### Immunoblot Analysis

Mouse kidney cortex tissue was homogenized in 5 volumes of protein lysis buffer (1% Triton X 100, 150 mM NaCl, 10 mM Tris-HCl, pH 7.4; 1 mM EDTA; 1 mM EGTA; 2 mM sodium orthovanadate; 0.2 mM phenylmethylsulfonylfluoride; 1 mM HEPES pH 7.6; 1 µg/ml leupeptin; and 1 µg/ml aprotinin) using a polytron homogenizer. The homogenate was stored on ice for 10 min and then centrifuged at 7,500 *g* for 5 min at 4°C. The supernatant was collected and protein determined using a bicinchoninic acid kit (Sigma Chemical Co, St. Louis, MO) with bovine serum albumin as the standard. Proteins (50–75 µg) were separated on 4–20% gradient sodium dodecyl sulfate polyacrylamide gels and transferred to nitrocellulose membranes. Membranes were blocked either in 5% dried milk or BSA in TBST (0.1% Tween 20 in 1× TBS) and incubated with 1∶1000 dilutions of anti-TGF-β_1_, anti-phospho-SMAD-3, anti-alpha-SMA, anti-phospho-p65, anti-ICAM-1 and anti-GAPDH overnight at 4°C. After incubation for 2 h at room temperature with secondary antibodies (1∶2000) conjugated with horseradish peroxidase, membrane proteins were detected by chemiluminiscence.

### Data and Statistical Analysis

Data are expressed as means ± SEM for all the experiments. Multiple comparisons of normally distributed data were analyzed by two-way ANOVA, as appropriate, and group means were compared using Bonferroni post-tests. Single comparisons were analyzed by Student’s *t*-test where appropriate. The criterion for statistical differences was *p*≤0.05 for all comparisons.

## Results

### Suramin had no Effect on Body Weights and Blood Glucose Levels

**Figure 1:**
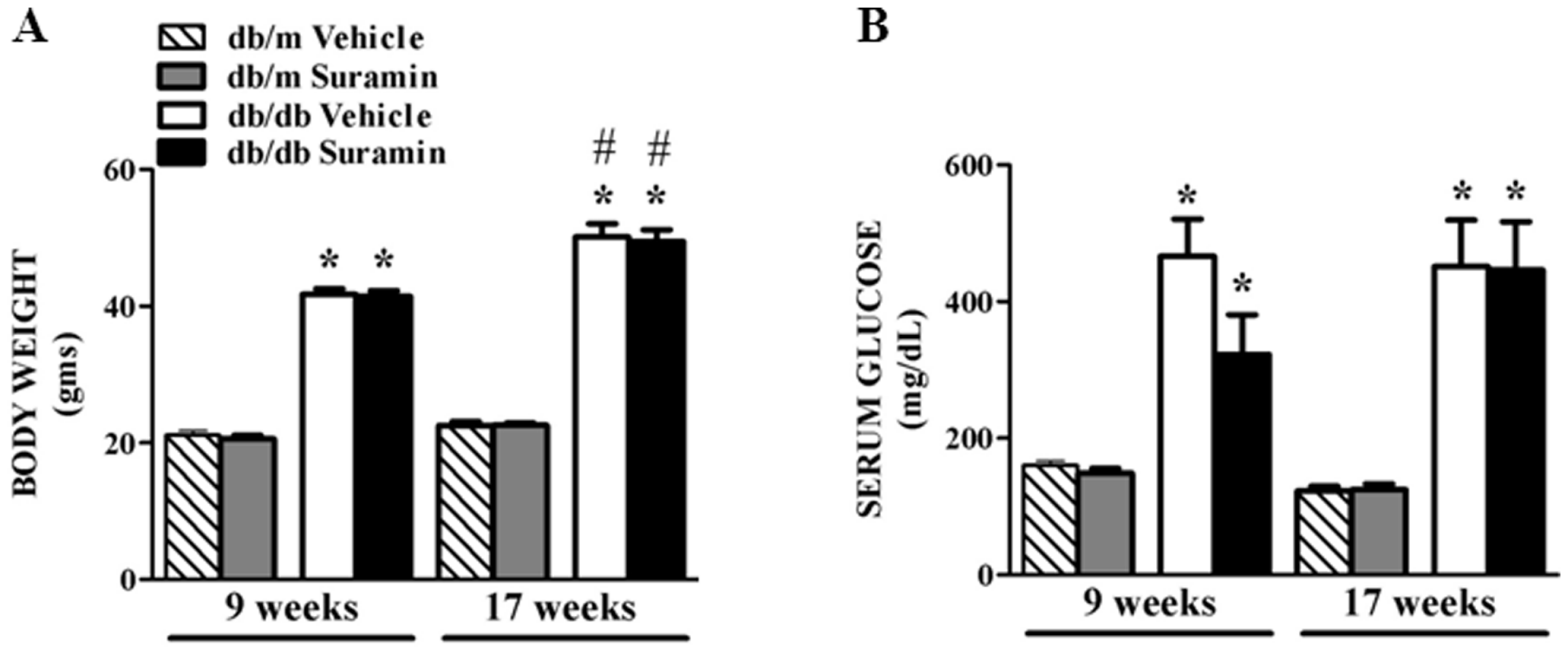
Effect of delayed administration of suramin on body weights and serum glucose levels in 9/17 week db/db mice. Non-diabetic *db/m* heterozygous mice of 8/16 weeks and diabetic *db/db* homozygous mice of 8/16 weeks, respectively, were treated with either saline vehicle or 10 mg suramin/kg (i.v., dissolved in saline). Mice in all groups were terminated a week later. (**A**) Body weights; and (**B**) Serum glucose levels; in 9 and 17 week non-diabetic and diabetic mice ± suramin intervention, respectively, were measured. Data are expressed as mean ± SE (n = 6–10). * Significantly different from respective non-diabetic *db/m* mice. (*p*≤0.05). **#** Significantly different from 9 week diabetic *db/db* mice. (*p*≤0.05).

8 and 16 week old *db/db* mice were chosen to simulate early and late stage T2DN, respectively, and the efficacy of suramin was tested in these mouse models. Suramin was given via a single intravenous injection and mice were sacrificed after one week. Body weights of 9 and 17 week old *db/db* mice ± suramin were greater than 9 and 17 week old *db/m* mice ± suramin, and the weight of 17 week old *db/db* mice was greater than 9 week old *db/db* mice (Fig. 1A). Serum glucose levels increased greater than 2.5-fold in 9 week old *db/db* mice ± suramin when compared to *db/m* ± suramin (Fig. 1B). Serum glucose levels did not further increase in *db/db* mice at 17 weeks. There were no differences in both body weights and serum glucose levels between 9 and 17 weeks old *db/db* mice ± suramin.

### Suramin Decreases Renal Proinflammatory Mediators

**Figure 2:**
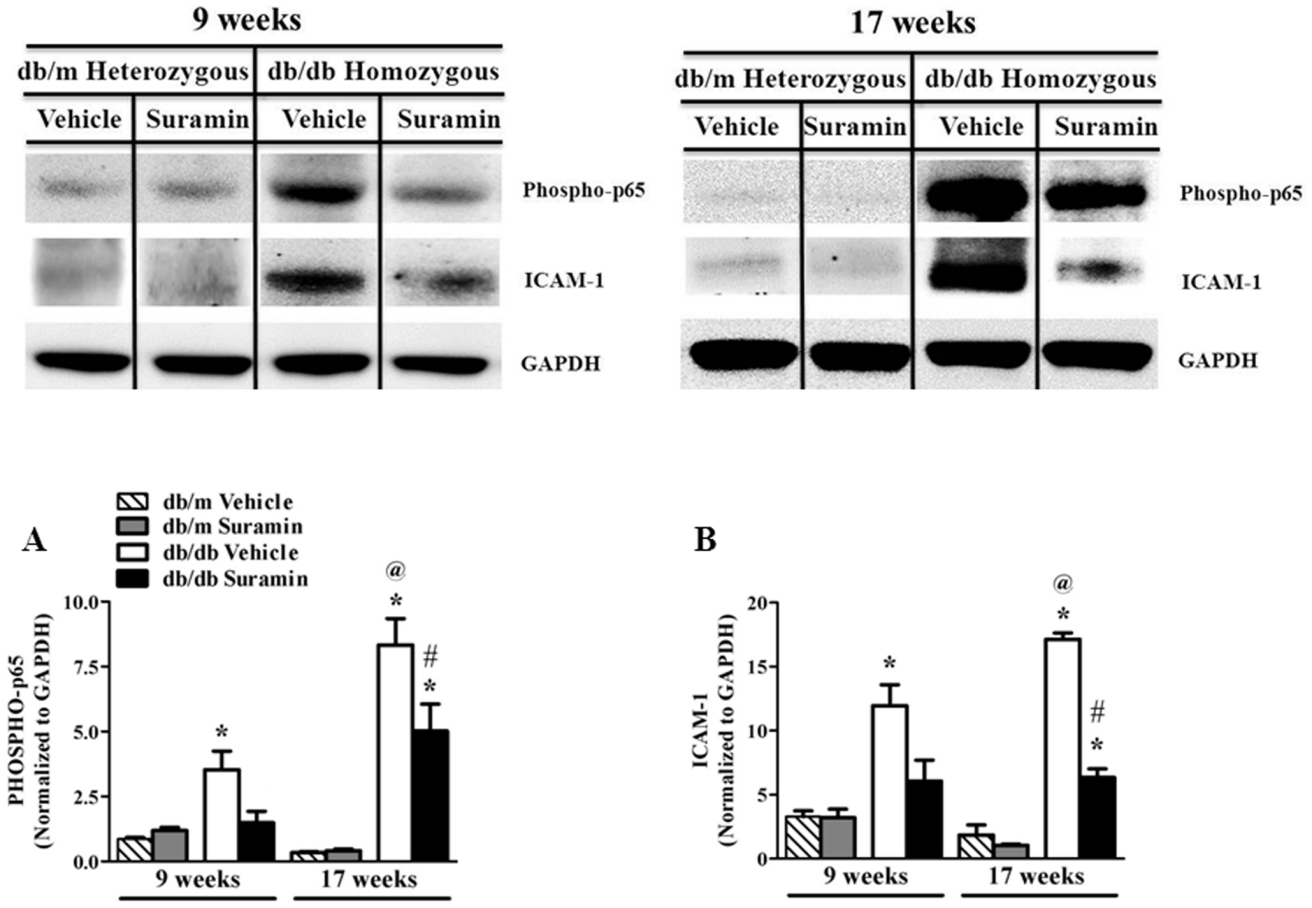
Effect of delayed administration of suramin on renal inflammation in 9/17 week db/db mice. Mice were treated as described in Figure 1. Representative Western blots showing inflammatory markers: phospho-p65 and ICAM-1 in kidneys of 9/17 week *db/db* mice ± suramin intervention. (**A**) Densitometric analysis of renal phospho-p65; and (**B**) renal ICAM-1. Data were normalized by GAPDH which served as internal control. Data are expressed as mean ± SE (n = 4). * Significantly different from respective non-diabetic *db/m* mice (*p*≤0.05). # Significantly different from vehicle-treated diabetic *db/db* mice (*p*≤0.05). @ Significantly different from vehicle-treated 9 week diabetic *db/db* mice (*p*≤0.05).

Hyperglycemia-induced activation of proinflammatory mediators like NF-κB is part of the pathogenesis of DN [7], [16], [17], [18], [19]. The active and proinflammatory form of NF-κB, phosphorylated-p65, increased in both 9 and 17 week old *db/db* mice when compared to 9 and 17 week old *db/m* mice ± suramin (Fig. 2A). Suramin intervention in 9 week old *db/db* mice decreased phosphorylated-p65 to control levels (Fig. 2A). Phosphorylated-p65 levels in 17 week old *db/db* mice were 2-fold higher than 9 week *db/db* old mice (Fig. 2A). There was a partial decrease in phosphorylated-p65 levels in 17 week *db/db* old following suramin intervention (Fig. 2A). Thus, a single dose of suramin reduced phosphorylation of NF-κB which is a proinflammatory factor in the progression of DN.

### Suramin Attenuates Renal ICAM-1 Expression and Decreases Leukocyte Infiltration

Activation of renal endothelial cell ICAM-1 and the contributions of leukocytes in the pathophysiology of renal inflammation in DN have been previously documented [20], [21], [22]. We found that ICAM-1 expression increased more than 3-fold in 9 week old *db/db* mice when compared to 9 week old *db/m* mice ± suramin (Fig. 2B). Suramin intervention in 9 week old *db/db* mice restored ICAM-1 levels to control levels (Fig. 2B). In the 17 week old *db/db* mice, we found a 9-fold increase in ICAM-1 protein expression when compared to 17 *db/m* mice ± suramin (Fig. 2B). Also, these levels were 1.4-fold higher than 9 week old *db/db* mice (Fig. 2B). Suramin intervention in 17 week old *db/db* mice resulted in a 2.7-fold decrease in renal ICAM-1 levels (Fig. 2B).

**Figure 3:**
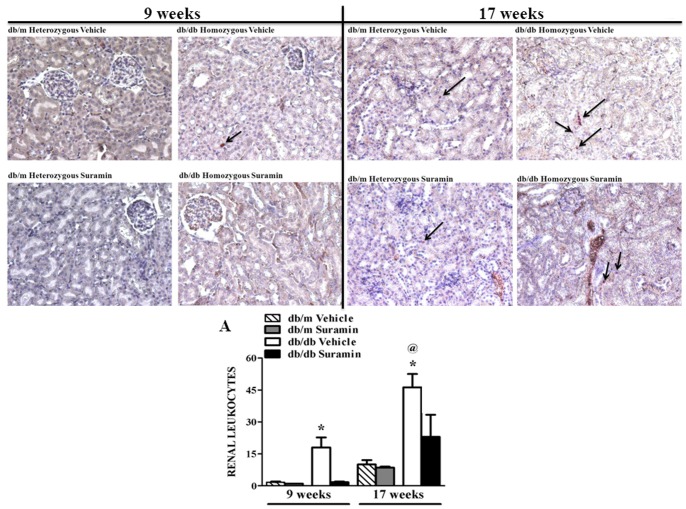
Effect of delayed administration of suramin on renal leukocyte infiltration in 9/17 week db/db mice. Mice were treated as described in Figure 1. Representative photomicrographs of neutrophil and monocyte staining assessed by formation of stable pinkish-red colored (arrows) complex of free naphthol and diazonium salts following incubation of kidney sections from 9/17 week non-diabetic and diabetic mice ± suramin intervention, respectively. All fields were chosen from the cortical regions of the kidney sections. Original magnification, 200 X. **A**. Quantitative analysis of renal leukocyte infiltration assessed by number of pink colored dots in a total of 25 fields in the cortical region of kidney sections. Data are expressed as mean ± SE (n = 4). * Significantly different from respective non-diabetic *db/m* mice (*p*≤0.05). @ Significantly different from vehicle-treated 9 week diabetic *db/db* mice (*p*≤0.05).

We also analyzed leukocyte infiltration in the corticomedullary region of the kidneys by staining neutrophils and monocytes with naphthol AS-D chloroacetate esterase as described previously [14], [19]. Renal leukocyte infiltration increased 18-fold and 5–fold in the 9 and 17 week old *db/db* mice, respectively, when compared to 9 and 17 week old *db/m* mice ± suramin (Fig. 3). It should be noted that renal leukocyte levels were higher in 17 week old *db/m* mice ± suramin when compared to 9 week old *db/m* mice ± suramin (Fig. 3). Renal leukocytes in 17 week old *db/db* mice were 2.5-fold higher than 9 week old *db/db* mice (Fig. 3A). Suramin intervention in 9 and 17 week old *db/db* mice restored renal leukocyte infiltration levels back to respective control levels (Fig. 3). Thus, suramin is effective in reducing the numbers of infiltrating leukocytes into the kidney.

### Suramin Decreases *TGF-β_1_/SMAD-3 signaling and fibrogenesis*

**Figure 4:**
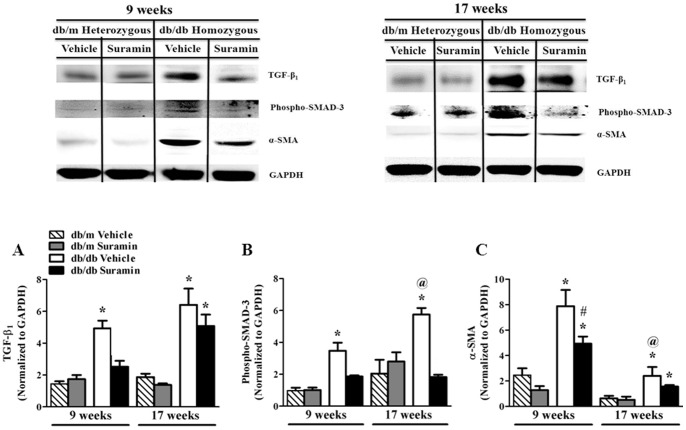
Effect of delayed administration of suramin on renal pro-fibrotic markers in 9/17 week db/db mice. Mice were treated as described in Figure 1. Representative Western blots showing profibrotic markers: Renal TGF-β_1_, renal phospho-Smad-3 and α-SMA in kidneys of 9/17 week *db/db* mice ± suramin intervention. (**A**) Densitometric analysis of renal TGF-β_1_; (**B**) Renal phospho-Smad-3; and (**C**) Renal α-SMA. Data were normalized by GAPDH which served as internal control. Data are expressed as mean ± SE (n = 4). * Significantly different from respective non-diabetic *db/m* mice (*p*≤0.05). # Significantly different from vehicle-treated diabetic *db/db* mice (*p*≤0.05). @ Significantly different from vehicle-treated 9 week diabetic *db/db* mice (*p*≤0.05).

The pathogenic role of TGF-β_1_ activation in hyperglycemia and increased accumulation of fibrogenic material during DN has been established [7]. Renal TGF-β_1_ protein was elevated 3.5-fold in 9 and 17 week old *db/db* mice when compared to 9 and 17 week old *db/m* mice ± suramin intervention (Fig. 4A). Suramin intervention in 9 week old *db/db* mice restored TGF-β_1_ levels back to control levels, but not in 17 week old *db/db* mice (Fig. 4A). We observed a 3-fold increase in SMAD-3 phosphorylation in 9 and 17 week old *db/db* mice, when compared to 9 and 17 week *db/m* mice ± suramin (Fig. 4B). Suramin intervention in 9 and 17 week old *db/db* mice restored SMAD-3 phosphorylation back to control levels (Fig. 4B). It should be noted that SMAD-3 phosphorylation in 17 week old *db/db* vehicle-treated mice was 1.7-fold higher than 9 week *db/db* mice (Fig. 4B).

TGF-β_1_/phospho-Smad-3 complex is known to translocate into the nucleus and regulate transcription of TGF-β_1_/SMAD-3-activated fibrogenic genes such as α-SMA and collagen 1A2 [7], [23]. Renal α-SMA protein expression increased more than 3-fold in 9 week old *db/db* mice when compared to 9 week old *db/m* mice ± suramin (Fig. 4C). Suramin intervention decreased α-SMA 30% in 9 week old *db/db* mice (Fig. 4C). While renal α-SMA protein expression increased in 17 week old *db/db* mice as compared to the *db/m* control mice, the overall level was much lower than in the 9 week old *db/db* mice and suramin intervention only partially decreased α-SMA expression in both 9 and 17 week *db/db* mice (Fig. 4C).

**Figure 5:**
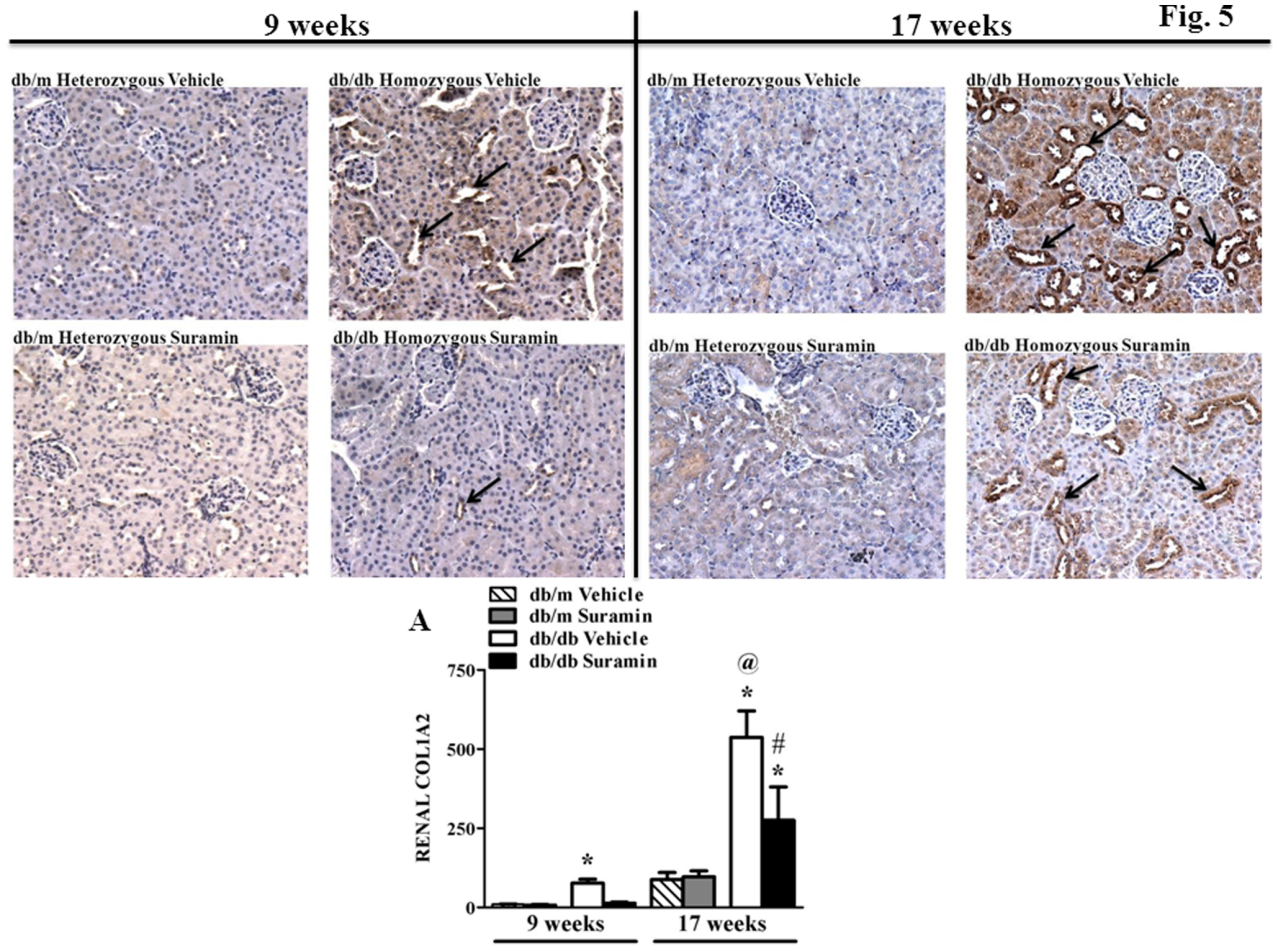
Effect of delayed administration of suramin on deposition of renal fibrogenic material COL1A2 in 9/17 week db/db mice. Mice were treated as described in Figure 1. Representative photomicrographs of COL1A2 deposition in kidney sections from 9/17 week non-diabetic and diabetic mice ± suramin intervention, respectively. All fields were chosen from the cortical regions of the kidney sections. Original magnification, 200 X. **A**. Quantitative analysis of renal COL1A2 assessed by brown staining in the epithelial cells lining the proximal tubules (arrows) in a total of 25 fields in the cortical region of kidney sections. Data are expressed as mean ± SE (n = 4). * Significantly different from respective non-diabetic *db/m* mice (*p*≤0.05). # Significantly different from vehicle-treated diabetic *db/db* mice (*p*≤0.05). @ Significantly different from vehicle-treated 9 week diabetic *db/db* mice (*p*≤0.05).

Kidneys from 9 and 17 week old *db/db* mice, respectively, exhibited a 7-fold increase in COL1A2 protein in the cortical region when compared to 9 and 17 week *db/m* mice ± suramin (Fig. 5A). Renal COL1A2 levels in 17 week old *db/db* mice were 7-fold higher when compared to 9 week *db/db* mice (Fig. 5A). Suramin intervention in 9 week *db/db* mice restored COL1A2 levels back to control levels (Fig. 5A). In addition, suramin treatment decreased COL1A2 deposition by 50% in 17 week old *db/db* mice.

### Suramin Decreases Renal Histological Changes

**Figure 6:**
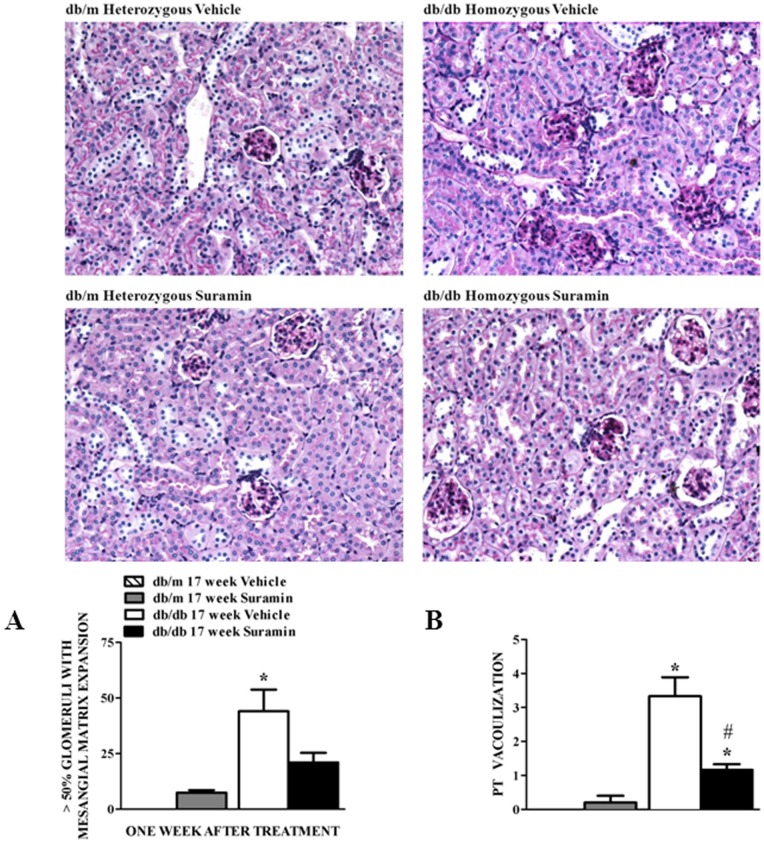
Effect of delayed administration of suramin on renal injury in 17 week db/db mice. Mice were treated as described in Figure 1. Representative photomicrographs of PAS-stained kidney sections from 17 week non-diabetic and diabetic mice ± suramin intervention, respectively. (**A**). Quantitation of glomeruli with more than 50% of their volume occupied by mesangial matrix in 17 week non-diabetic and diabetic mice ± suramin intervention. (**B**) Quantitation of proximal tubules with vacuolization in 17 week non-diabetic and diabetic mice ± suramin intervention. Data are expressed as mean ± SE (n = 6). * Significantly different from respective non-diabetic *db/m* mice. (*p*≤0.05). **#** Significantly different from vehicle-treated diabetic *db/db* mice. (*p*≤0.05). **PT**, Proximal tubule. All fields were chosen from the cortical regions of the kidney sections. Original magnification, 200 X. It should be noted that we could not detect any glomeruli with mesangial matrix expansion or proximal tubules with vacuolization in the vehicle-treated non-diabetic *db/m* control mice kidneys.

Since suramin reduced markers of inflammation and fibrosis in 9 and 17 week old *db/db* mice, we determined whether suramin improved renal pathology in 17 week old *db/db* mice which represents advanced stage of T2DN exhibiting renal inflammation and fibrosis usually seen in clinical cases of human DN [8]. No detectable glomerular changes are seen in the vehicle-treated *db/m* mice. Bowman’s capsule was of usual caliber with no evidence of epithelial cell proliferation, the capillary tuft was fully expanded with patent capillary loops, and the glomerular basement membrane (GBM) was thin and delicate. The mesangium contained the usual complement of cells and matrix without matrix expansion, inflammation, or sclerosis (Fig. 6). In contrast, *db/db* mice had larger glomeruli with increased mesangial matrix. The mesangial matrix and glomerular enlargement were more pronounced and thickening of the GBM was notable. Nodular lesions of the subepithelia basal lamina were also noted. Morphometric analysis revealed glomeruli with greater than 50% of glomerular volume filled with mesangial matrix increased (Fig. 6A). The glomerular capillary basement membranes appeared thickened; the mesangium was diffuse and expanded with PAS-positive matrix material. The overall cellularity was normal without inflammation or necrosis. Thus, by light microscopy, the appearance of glomeruli is very similar to glomeruli observed in human DN [8]. There was no indication of glomerulosclerosis in any of the kidneys.

No detectable proximal tubular changes are seen in the vehicle-treated *db/m* mice. However, tubular changes in the *db*/*db* mice primarily consisted of vacuolization of proximal tubular cells (Fig. 6B). There was also the presence of arteriolar hyalinosis, vacuolization of the distal segment, PAS positive material in distal segment cells/lumen and PAS positive droplets in proximal tubules. There was no evidence of tubular atrophy, tubulointerstitial fibrosis, or alterations of the medullary structure (Fig. 6).

Interestingly, delayed administration of a single dose of suramin intervention reversed these glomerular and tubular lesions in 17 week old *db/db* mice (Figs. 6A & B). There was a marked decrease in glomerular matrix expansion and the number of glomeruli with severe deposition of matrix material had dropped to basal levels (Fig. 6A). Proximal tubular vacuolization was partially reversed after suramin treatment (Fig. 6B). Thus, in this 17 week old *db/db* mouse model, delayed administration of a single dose of suramin reversed many pathological lesions that are hallmark features of DN.

### Suramin Improves Renal Function

**Figure 7:**
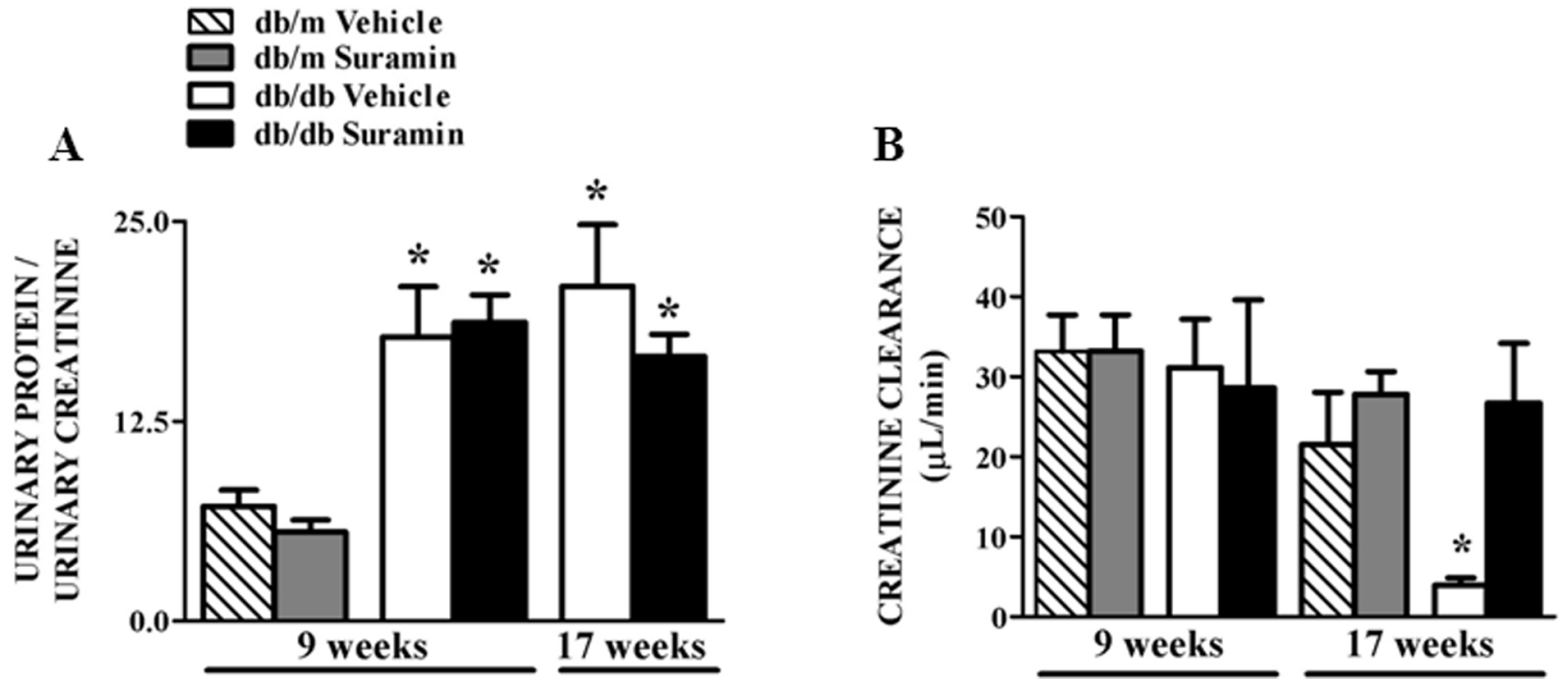
Effect of delayed administration of suramin on urinary protein excretion and creatinine clearance (CrCl) in 9/17 week db/db mice. Mice were treated as described in Figure 1. Urine was collected on ice 24 h before euthanization and (**A**) Urinary protein excretion normalized to urinary creatinine; (**B**) Creatinine clearance; in 9 and 17 week non-diabetic and diabetic mice ± suramin intervention, respectively, were measured. Data are expressed as mean ± SE (n = 3–6). * Significantly different from suramin-treated diabetic *db/db* mice. (*p*≤0.05).

Since renal inflammation, fibrosis (in both 9 and 17 week *db/db* mice) and pathology (17 week *db/db* mice) were improved with suramin, we determined if this translated to improved renal function. 9/17 week *db/db* mice ± suramin had significantly higher urine output (9 week *db/db* mice vehicle: 1.6±0.2 ml, 9 week *db/db* mice suramin: 1.4±0.2 ml; 17 week *db/db* mice vehicle: 1.3±0.5 ml, 17 week *db/db* mice suramin: 1.3±0.4 ml) when compared to 9/17 week *db/m* mice ± suramin. Total urinary protein normalized to urine creatinine increased 2.5-fold in 9 and 17 week old *db/db* mice ± suramin when compared to *db/m* mice ± suramin (Fig. 7A). Suramin had no effect on 24 h urine output and urinary protein/urinary creatinine values in 9 or 17 week *db/db* mice. Creatinine clearance (CrCl) in 9 week old *db/db* and *db/m* mice ± suramin was equivalent. However, CrCl in 17 week *db/db* mice decreased greater than 80% when compared to *db/m* mice ± suramin and suramin restored CrCl to control levels (Fig. 7B). Thus, renal function was decreased at 17 weeks in *db/db* mice and was restored by suramin.

## Discussion

We chose the leptin receptor-deficient diabetic *db/db* mouse because it is extensively used to investigate T2DN and mimics many of the effects observed in human DN [8], [9], [10]. Furthermore we chose to examine diabetic changes at 9 and 17 weeks of age because these time points represent the early and advanced stage of DN, respectively. Overall our data reveal that in the kidneys of 9 week old *db/db* mice the proinflammatory molecules NF-κB and ICAM-1 are elevated; leukocytes have infiltrated; and profibrotic factors TGF-β1/pSMAD-3, COL1A2 and α-SMA are increased. Associated with these changes at 9 weeks is an increase in urinary protein excretion without a decrease in CrCl. While the renal changes described above are generally maintained or increased further (see details below) at 17 weeks of age, CrCl is decreased and there are marked changes in renal pathology. Therefore, these renal changes represent the early and late changes in DN.

Our objective for the current study was to determine if delayed administration of a single dose of suramin attenuates renal inflammation and fibrosis during the early and late stages of DN. Impressively, one week following a single administration of suramin in 9 and 17 week *db/db* mice, NF-κB, ICAM-1, leukocyte infiltration, TGF-β1/pSMAD-3, COL1A2 and α-SMA were all decreased or returned to control levels. Furthermore, renal pathology improved and CrCl returned to control levels in the 17 week old mice with DN. These results provide strong evidence that suramin therapy may be an additional approach in the treatment of DN.

Angiotensin converting enzyme inhibitors and angiotensin II receptor blockers have become the standard of care in diabetic patients with microalbuminuria. However, increasing evidence suggests that these agents are not completely efficacious in slowing the progression of DN [24], [25]. Suramin may provide an alternate/additional approach in the treatment of DN. Suramin has a known safety profile and because its plasma half-life is long (44–54 days) and it accumulates mostly in the kidney [13], [26] weekly or longer dosing periods are possible.

Moreover, delayed administration of one dose of suramin was recently shown to block early activation of proinflammatory and profibrotic signaling events in an animal model of early Type 1 diabetes-induced kidney disease [19]. In the current study, sustained effects of one dose of suramin effectively decreased proinflammatory and profibrotic factors in both 9 and 17 week *db/db* mice along with marked improvement in renal function and pathology in 17 week *db/db* mice [8].

Among several mouse models of diabetes that have been identified, the *db*/*db* mouse appears to most closely mimic the progressive nature of mesangial matrix expansion seen in human DN [8]. Seventeen week old diabetic mice exhibit the hallmark changes of mesangial matrix expansion and proximal tubular epithelial cell vacuolization [8], whereas these changes are not observed in 9 week old mice kidneys [8]. Surprisingly, one dose of suramin restored renal function and decreased these pathological lesions in the 17 week old mice. This is a unique observation that, at least in this *db/db* mouse model, the renal changes are reversed within one week and that a continuous injury to drive DN has been blunted.

Proteinuria is a modifiable risk factor for the progression of renal disease [27], [28], [29]. However, the cumulative incidence of ESRD was shown to be very high when there was a low CrCl with proteinuria [29]. In our study, we observed that 17 week *db/db* mice had low CrCl with proteinuria and suramin intervention in 17 week *db/db* mice restored CrCl to control levels, indicating suramin might prevent development of DN to ESRD. Also, increased urinary albumin excretion before the development of obvious structural evidence of alterations of the GBM or the podocytes was noticed in 8 week *db/db* mice [8]. The lack of an effect of suramin on protein excretion in 9 and 17 week *db/db* mice suggests that suramin does not elicit its actions by targeting neither podocyte numbers nor podocyte-specific proteins in relation to the development of albuminuria in the *db*/*db* mouse. Previous reports suggest that at 8 week of age, there is a two-fold increase in CrCl in *db*/*db* mice when compared to *db/m* control mice [8]. This finding suggests that these *db*/*db* mice might be hyperfiltering at the onset of hyperglycemia and that GFR declines with the duration of diabetes [8], [30]. However, in our hands we found CrCl in 9 week *db/db* mice was similar to controls ± suramin intervention. Whereas, in 17 week old *db*/*db* mice, the CrCl was decreased compared to healthy *db/m* mice ± suramin as previously observed [8], [30] and one dose of suramin restored renal function back to normal. Thus, the pattern of CrCl associated with the duration of diabetes in the *db*/*db* mouse corresponds with the actual GFR levels reported by Gartner [31]. It should be noted that the improvement in renal histology and function is not due to changes in serum glucose levels, but rather the effect of suramin on the kidney.

Nuclear translocation of NF-κB (phosphorylation of p65 subunit) in endothelial cells caused by hyperglycemia and oxidative stress has been linked to transcriptional activation of adhesion molecules like ICAM-1 [32]. Circulating leukocytes use endothelial cell ICAM-1 as an anchor to transmigrate and cause inflammation [33]. Activation of renal endothelial cell ICAM-1 expression by hyperglycemia and subsequent leukocyte infiltration has been suggested to be an important event in pathophysiology of inflammation in DN [20], [22], [34], [35]. We observed that suramin intervention after DN decreased phosphorylation of p65, blocked ICAM-1 expression and decreased leukocyte infiltration. However, a single dose of suramin did not return NF-κB to control values in 17 week old *db/db* mice when compared to 9 week old *db/db* mice, and this might also lead to the partial decrease in ICAM-1 in 17 week old *db/db* mice.

The anti-purinergic or anti-G-protein-coupled receptor effects of suramin may be responsible for the observed anti-inflammatory effects seen in 9/17 *db/db* mice following intervention. The functional role of activated GPCR-mediated purinergic signaling in inflammatory diseases and the contribution of disordered purinergic signaling to the mechanisms of acute and chronic diseases is established [36]. For example, purinergic receptor activation is known to increase nuclear translocation of transcription factors like NF-κB and elicit inflammatory response [37].

Therefore, mechanistically these sequential events may result from the anti-inflammatory actions of suramin exerted either by antagonizing P2 receptors or by a direct inhibitory effect of suramin on NF-κB activation as previously reported [19], [36], [38].

High-glucose is also known to activate transforming growth factor-β_1_, which is widely thought to be the most pertinent cytokine in fibrotic renal pathology typically observed in patients with chronic progressive DN [7]. When stimulated, TGF-β_1_ activates the TGF-β_1_ receptor, SMAD-3 is phosphorylated and the resulting complex translocates into the nucleus and regulates transcription of TGF-β_1_ target fibrogenic genes such as α-SMA, collagen 1A2 and fibronectin-1 [23]. It has been shown previously, that pharmacological inhibition of TGF-β_1_ using a 12 week dosing regimen, prevents renal hypertrophy, mesangial matrix expansion, increase in collagen and fibronectin expression, and decline in renal function in *db/db* mice [39], [40]. Our study demonstrates the anti-fibrotic potential of a single dose of suramin after the establishment of progressive DN.

Suramin was previously suggested to inhibit fibrotic and growth inhibitory actions of TGF-β_1_ and inhibit renal fibrosis [13], [15], [19], [41]. In our study, we found that renal TGF-β_1_ and phospho-SMAD-3 protein expressions were elevated in 9 and 17 week old diabetic mice when compared to respective non-diabetic control mice and this increase was more exaggerated in 17 week than 9 week diabetic mice. While one dose of suramin decreased TGF-β_1_ in 9 week diabetic mice, it did not decrease renal TGF-β_1_ in the 17 week old *db/db* mice, which is likely due to the advanced stage of DN and extent of damage. We observed that suramin inhibited renal TGF-β_1_– mediated activation of SMAD-3 in the 9 and 17 week diabetic mice, supporting our previous finding of the anti-fibrotic potential of suramin in early hyperglycemia-induced kidney injury. Interestingly, phosphorylation of SMAD-3 was lowered in *db/db* mice at 17 weeks suggesting a different mechanism of action.

In this regard, adenosine receptor subtypes (especially A2A and A2B subtypes), have multiple roles in inflammation and fibrotic pathways. For example, activation of A2A receptors suppress the deposition of collagen types I and III, reduced the infiltration of CD4+ T lymphocytes, and attenuated the expression of TGF-β1, which in turn inhibited inflammation and renal interstitial fibrosis in an obstructive nephropathy model [42]. In contrast, use of an antagonist of the A2B receptor subtype blocked TGF-ß_1_-mediated fibrosis in an animal model of chronic kidney disease [43]. Therefore, it is unlikely that suramin exerts its anti-inflammatory and anti-fibrotic effects by inhibiting A2A receptors. It is plausible that suramin may inhibit A2B receptor activation and exert an anti-fibrotic action. α-SMA–positive myofibroblasts, which are the principal effector cells responsible for ECM overproduction in the fibrotic kidney along with profibrotic proteins like fibronectin-1 and collagen 1A2 [44], were largely decreased by suramin in 9 week diabetic mice. However, a single dose of suramin did not decrease α-SMA protein in 17 week diabetic mice, which could be due to the exaggerated fibrotic response in these kidneys. A more reliable and consistent marker of renal fibrosis is COL1A2. We observed that suramin decreased deposition of renal COL1A2 in both 9 and 17 week old diabetic mice. Interestingly, we did not see COL1A2 staining in interstitial cells. This was unexpected since it was anticipated that COL1A2 accumulation would occur mainly in interstitial fibroblasts. This suggested to us that in this mouse model of 17 week T2DN, fibroblasts might not participate in COL1A2 synthesis. This finding raised the question as to whether COL1A2 is transcribed in epithelial cells of these mice with well-established DN. This also indicates that the inflammatory responses might re-programme epithelial cells to transcribe COL1A2. This hypothesis was supported by recent studies in other renal fibrotic diseases like aristolochic acid-induced renal fibrosis [45] and in kidney fibrosis using human biopsy samples [46]. One other possible explanation could be that kidney pericytes have been reported to play an important role in fibrosis and type I collagen transcription previously [47].

The finding that epithelial cells participate in collagen transcription is novel and provides an alternative explanation for the source of interstitial collagen accumulation during DN. Also, it has long been proposed that injured tubular epithelial cells undergo epithelial mesenchymal transition (EMT) to give rise to a proportion of the activated interstitialmyofibroblasts in renal fibrosis [48]. Whether EMT really happens in kidney fibrosis in this T2DN model is not clear. Collectively, the data suggest that suramin acts upstream of TGF-β_1_-SMAD-3 signaling and blocks this fibrotic pathway.

In conclusion, this work advances the field and is translational by demonstrating a potential new therapy for DN.
