# Correction: Suramin: A Potential Therapy for Diabetic Nephropathy

**DOI:** 10.1371/journal.pone.0114426

**Published:** 2014-11-19

**Authors:** The PLOS ONE Staff

The second author's name is spelled incorrectly. The correct name is: Lauren A. Howell. The correct citation is: Korrapati MC, Howell LA, Shaner BE, Megyesi JK, Siskind LJ, et al. (2013) Suramin: A Potential Therapy for Diabetic Nephropathy. PLoS ONE 8(9): e73655. doi:10.1371/journal.pone.0073655

The Author Contributions should read: Conceived and designed the experiments: MCK LJS RGS. Performed the experiments: MCK LAH BES. Analyzed the data: MCK LAH JKM LJS RGS. Wrote the paper: MCK LJS RGS.
